# Eyes and Sight

**Published:** 1889-01-12

**Authors:** 


### The Hospital

A Weekly Institutional Journal of

Science, Medicine, Nursing, and Philanthropy.

*For Hospitals, Asylums, Medical Practitioners, Students, Nurses, Families, Parishes, Congregations, and the Charitable Public.*

Vol. V. No. 120.] [January 12, 1889.

### Eyes and Sight.

The eyes may be called the most "intelligent" of all the senses. They have not only the largest range but the clearest understanding of many and varied objects. Compare sight, for example, with hearing, smell, taste, or touch, and it is seen at once that the eyes have a more comprehensive, immediate knowledge than all the other senses put together. Imagine a world of blind men, a ball-room of blind women, an army of blind soldiers, or, what would be much more pathetic, a large infant school full of blind children. It is clearly impossible to exaggerate the value and importance of the sense of sight. What the mind is to any individual mental faculty such as music, logic, or mathematics, that in a measure is the eyesight to all the minor and less indispensable senses. Nothing better indicates the general progress of the civilised races than the eagerness with which intelligent men and women try to measure their environment and and their natural fitness to cope with it. All the more rational of human beings stand in the midst of their affairs, not as the primeval savage used to do, with a feeling of overwhelming awe, terror, and powerlessness, but rather with the mind and instincts of conquerors who have determined by the adaptation of means to ends, by the strengthening of that which is weak in themselves and in their circumstances, by the avoidance of that which is obstructive and dangerous, and by all the helps which science and natural intelligence bring to their aid, to be victorious in life's struggle. A soldier carries an equipment of various arms, but everyone will admit that his rifle is his principal weapon of offence or defence. With that he can slay or disable at a distance as well as at hand. What the rifle is to the soldier that eyes are to men, women, and children, and very much more. What a miserable soldier is he who does not care whether his rifle is in good order and clean or dirty, out of repair, and useless! Such soldiers never lead armies or conquer countries. They are always dullards to whom time gives no rewards except whips and scorns, contempt and poverty. But those who, having any defect or peculiarity of vision, disregard it, and make no efforts to have it removed or at least explained, must be considered to be on a level with the soldier who cares nothing though his rifle is broken and with the workman who is heedless that his tools are out of date and useless.

Not very long ago the average doctor knew little or nothing about the eyes. He did not even know their natural structure, much less their defects and their diseases, and how to cure them. Medical men in middle life often call to mind, with a feeling of profound shame, the ignorant, helpless, and ruinous blundering which used to characterise the treatment of eyes by some ordinary practitioners. Many a child, and even grown-up person, suffering with eye affections, which, if left to nature, would often have disappeared of themselves, has been taken in hand by a conscienceless practitioner, and blinded without hope of remedy. Happily those days are now past. Not only has the specialist come in with his knowledge and skill, but the average practitioner has learnt to know and to understand that the eye is an organ which cannot be maltreated without imminent risk; and therefore it is his custom, on the slightest evidence of any disease which is outside the limits of his experience, to pass the case on at once to the oculist. This is entirely as it should be. No man has a right to treat any case which he does not understand, still less the diseases of any organ in which he has had little or no training at all.

The peculiarities and defects of eyesight are very numerous in what may otherwise be called normal or non-diseased eyes. Some children, for example, are born short-sighted; others have, perhaps, an opposite condition. Some are born with a double squint, some with an upward and others with a lateral turn of the eyeball. All these conditions it was formerly the fashion to consider hopeless. Among the better classes, at any rate, they are now invariably attended to; but among the poor they are still, to a large extent, left to nature. There is, however, no excuse for this in these days; and particularly in large towns. Parents who see any visible deformity in the eye, or who notice their children holding up a book close to their noses, or fixing and screwing up the muscles of the face in some unnatural way to see objects that are at all distant, should use the little intelligence that is required to tell them that something is wrong. Then they can at least take their little ones to the eye department of the nearest hospital, where, if the case can be improved or cured, it will be done, and the child made to that extent not only more presentable, but fitter for the battle of life. We need not point out for the thousandth time how many recruits are rejected for the Army, and the still larger number that are declined for the Navy, on account of some defect of vision; it may be short sight, or colourblindness, or any other disabling peculiarity. But these rejections give us a distinct indication of the proportion of the population whose eyes cannot be called perfect. Parents are not to be forgiven who, from mere idleness and inattention, allow their children to grow up with a defect which will always cause inconvenience, and will not seldom be the one single bar to an otherwise prosperous career.

It is not sufficiently understood that not only may races of men be improved, but that particular organs in individual men may be made fitter for their functions. Of course no one proposes at the present time to set to work to improve every detail of the ear, nose, tongue, finger, foot, and so on; although if anyone should do so he would be distinctly a benefactor to his kind. If a sufficient measure of physiological knowledge could be spread abroad, and an adequate degree of what may be called physiological enthusiasm could be imparted to a large number of the population to establish a "Cult" for the anatomical and physiological improvement of the race, that Church would be much better worthy of the consideration of intelligent religionists than many faiths that have played no inconsiderable part in the world's history. For the present, however, we spare our readers that, and confine ourselves to this single article of physiological faith, viz., that the sight of the people both can be and ought to be steadily improved. It is obvious that we cannot here go into details, but we do reiterate this first lesson in the art of eye improvement—that every man, woman, and child who has any peculiarity should submit himself to the inspection of an honest and competent oculist. With regard to those who have no such peculiarity it is obvious that a little intelligence will enable them to think out ways of improving their sight themselves; and at least, if they do not enter upon a course of sight improvement, they may be warned against those common practices which tend to injure vision. Surely no grown up man will nowadays, for example, read with insufficient light, still less will he write, or draw, or paint. But the mere avoidance of injuries to the eye should not be sufficient for those who desire to preserve in the utmost perfection this all-important and beautiful organ.

The conclusion of the whole matter is this: that, as is usually the case, no one can help us so well as we can help ourselves. Any man who has no decided defect of sight has within his own hands the power of improving and preserving it. At the foundation of such preservation and improvement lies good general health. Next in importance to this, of course, is the judicious use of the eyes. They should be taught to see all kinds of objects, great and small, at all kinds of distances. They should not be employed for too long a time upon any particular class of objects, but should be used like other organs to the highest degrees of their power and endurance, but without fatigue. When the time comes that they are no longer able to meet the demands made upon them of themselves, then artificial aid should be sought in the shape of suitable glasses. These directions are as obvious as they are elementary; but the main purport of this article will be missed it we do not enter a special plea on behalf of those who cannot help themselves. Children of all ranks are at the mercy of their parents; and although parents may not be unkind or unjust they may be excessively stupid, and their stupidity may bring about as much harm as unkindness or injustice. If any readers have themselves children who show visual peculiarities of any kind, however slight, let them not lose a moment before placing them under proper care. If they have no children themselves, they may have friends whose little ones are thus disabled and deformed; then they should by all means make known to them the risks which are in store. In a world of plenty it is pitiful that any should hunger; in a world of beauty it is sad that any should fail to see all that may be seen.

